# Commentary on ‘Will vaccination refusal prolong the war on SARS-CoV-2?’

**DOI:** 10.1136/postgradmedj-2020-139043

**Published:** 2020-10-28

**Authors:** Philip D Welsby

**Affiliations:** Retired, Edinburgh, UK

There are four ways to combat COVID-19. First, physical interventions including mask-wearing and social distancing but ultimately including lockdowns that carry huge social, psychological and economic costs. Second, vaccines, none of which at the time of writing (September 2020) are known to be effective and safe. Third, drugs active against COVID-19 but the only effective drugs, remdesivir and dexamethasone, are only used in life-threatening infections. Fourth, administration of convalescent sera, the efficacy and safety of which are being ascertained.

The authors report that only 58% of those surveyed would be willing to be vaccinated, 16% were neutral and 26% were not planning to be vaccinated. A similar US study reported a 67% acceptance of a COVID-19 vaccine with noticeable demographic and geographical disparities in vaccine acceptance.^1^ It seems likely that the probable vaccine refusal rate will be about 25% and, if so, that it is possible that a vaccine alone may not be the solution unless measures are undertaken to counter vaccine refusal. Even if a vaccine were available by the end of the year, governments would have to decide whether it was the vaccine that should be rolled out across whole populations.

An overview of coronavirus vaccine status reported 139 preclinical vaccines were not yet in human trials, 25 were in small-scale safety trials, 15 were in expanded safety trials and none, apart from Russia’s ‘Sputnik’ vaccine, have been approved for general use. Thus, there will be several vaccines, some of which will be promoted because of political considerations that might well pre-empt safety considerations. President Putin’s introduction of the Russian ‘Sputnik’ vaccine is an example. Some other governments, especially popularist governments, will tell the people what they think they want to hear and may roll out their vaccine prematurely.

Global pandemics require a global solution, and the independent development of their own vaccines by various countries’ ‘vaccine nationalism’ should be deplored. Sadly, it seems that nationalistic tendencies include undermining of the potential of the WHO.

There is a widespread belief that the third of these, vaccination, will solve the COVID-19 pandemic. Indeed, this commentator had similar views before reading this and similar surveys of vaccine acceptance rates.

To achieve high vaccination rates, most vaccinees would need to know that the vaccine in question conferred significant protection *to them as individuals* especially as some will not realise their vaccination would also protect others including those who cannot be vaccinated. Vaccine refusers as surveyed may have some justification. The survey respondents almost certainly did not realise the range of vaccines being developed, how effective each might be for them *as individuals* and for others in their society, how long immunity might last and potential side effects. It would be interesting to know if vaccine refusers came from sections of the community who are least likely to develop significant illness from COVID-19 infection and if they were also those most likely to ignore official advice about risk minimisation. Vaccine refusal and disregard of risk minimisation could be an extremely dangerous combination.

Vaccine refusal, as the authors point out, may be a very significant problem. This paper^2^ is a survey of surveys and not all the constituent surveys would have been similar such that they may not be representative of the populations studied. To combine such surveys may be considered statistically inappropriate, but the results cannot be ignored.

Predicting the protection offered by each candidate vaccine in various population groups will be necessary, especially if herd protection is also an aim. Would multiple doses be required? What would be the risks of each vaccine especially if vaccine introduction were rushed? One example of a rushed vaccine introduction was President Ford’s vaccination of 25% of the US population against swine influenza because of the Fort Dix outbreak in 1976 that caused a fourfold increase in Guillain-Barré syndrome.

Without doubt, the authors are correct that vaccine refusal needs to be minimised, but the question must be asked ‘Will the uptake rate of vaccination alone be sufficient to ensure that the COVID-19 pandemic is solved or at least minimised?’ If the answer is ‘No’, then we also need to concentrate more effort into developing drugs that make any symptomatic COVID-19 infection trivial. It then would then be of much less import if COVID-19 persisted at vaccine-induced low levels (it is likely that the time required for assessment of such drugs would be shorter than that for vaccination). Normal life could resume. The surprisingly small number of such drugs has been summarised.^3^

What a pity a vaccine had not been developed against coronavirus common colds. We might have had an easily modified vaccine in waiting.
